# The application of thermophilic DNA primase TtDnaG2 to DNA amplification

**DOI:** 10.1038/s41598-017-12241-6

**Published:** 2017-10-09

**Authors:** D. Zhao, Xiuqiang Chen, Kuan Li, Yu V. Fu

**Affiliations:** 1 State Key Laboratory of Microbial Resources, Institute of Microbiology, Chinese Academy of Sciences (CAS), Beijing, 100101 China; 2 Savaid Medical School, University of Chinese Academy of Sciences, Beijing, 100101 China; 3 State Key Laboratory of Mycology, Institute of Microbiology, Chinese Academy of Sciences (CAS), Beijing, 100101 China

## Abstract

For DNA replication *in vivo*, DNA primase uses a complementary single-stranded DNA template to synthesize RNA primers ranging from 4 to 20 nucleotides in length, which are then elongated by DNA polymerase. Here, we report that, in the presence of double-stranded DNA, the thermophilic DNA primase TtDnaG2 synthesizes RNA primers of around 100 nucleotides with low initiation specificity at 70 °C. Analysing the structure of TtDnaG2, we identified that it adopts a compact conformation. The conserved sites in its zinc binding domain are sequestered away from its RNA polymerase domain, which might give rise to the low initiation specificity and synthesis of long RNA segments by TtDnaG2. Based on these unique features of TtDnaG2, a DNA amplification method has been developed. We utilized TtDnaG2 to synthesize RNA primers at 70 °C after 95 °C denaturation, followed by isothermal amplification with the DNA polymerase *Bst*3.0 or phi29. Using this method, we successfully amplified genomic DNA of a virus with 100% coverage and low copy number variation. Our data also demonstrate that this method can efficiently amplify circular DNA from a mixture of circular DNA and linear DNA, thus providing a tool to amplify low-copy-number circular DNA such as plasmids.

## Introduction

DNA replication is a complicated process *in vivo* ^1–3^. It requires numerous components working together to synthesize new DNA strands. Since no known replicative DNA polymerase can initiate DNA synthesis *de novo*, primers that are synthesized by a primase are required to provide a free 3′-hydroxyl group for DNA polymerase.

Primases can be divided into two different families: Archaeal Eukaryotic Primase (AEP)-like and DnaG-like^4,5^. The archaeal–eukaryotic primases belong to the AEP superfamily, and they usually consist of two different units: a catalytic unit and an accessory unit. Human PrimPol (cccdc111) has been reported to have both polymerase and primase activity^6^. In contrast, prokaryotic primases are usually monomeric proteins, belonging to the DnaG superfamily^5^. *Escherichia coli* primase DnaG has been well studied. It consists of three conserved domains: N-terminal zinc binding domain (ZBD), central RNA polymerase domain (RPD) and C-terminal helicase interaction domain (DnaB-ID)^1,7,8^. The RPD is responsible for RNA primer synthesis, the DnaB-ID interacts with helicase, and the function of ZBD is modulating the specificity of DNA binding sites and primer length^8,9^. In bacteria, primases associate with DNA helicases to form a complex called the primosome. DnaG binds to the replicative helicase DnaB, making the helicase–primase complex much more active than DnaG alone^10,11^. Like DnaG, most virus primases are also monomeric proteins. The gene 4 protein (gp4) from T7 phage serves as both primase and helicase^12^, but T4 primase (gp61) binds tightly to T4 helicase (gp41) to form a primosome^13^. In most if not all primases analysed to date, primer synthesis is not initiated completely at random on a template. For example, the T7 gp4 prefers 3′-CTGG(G/T)-5′ and 3′-CTGTG-5′ to initiate synthesis^14^. In contrast, the *E*. *coli* DnaG recognizes 3′-GTC-5′ and generates short RNA primers for DNA synthesis^15^, while the human Ccdc111 primase shows strong preference for 3′-GTCC-5′ sequences^6^.

TtDnaG2 is a primase discovered in the thermophilic bacterium *Thermoanaerobacter tengcongensis* MB4. Purified TtDnaG2 can efficiently synthesize RNA primers on a single-stranded DNA template *in vitro* at a wide range of temperatures from 37 °C to 85 °C^16^. In contrast to most primases, TtDnaG2 exhibits extremely loose template specificity; for example, it was shown to initiate RNA synthesis at all of the templates tested in a previous study^16^. Another important feature of TtDnaG2 is the ability to synthesize remarkably long RNA primers (>100 nucleotides). This contrasts markedly with *E*. *coli* DnaG and T7 gp4, which can only synthesize 10–12- and 4–5-nucleotide-long RNA primers, respectively^15^.

*In vitro* amplification of genomic DNA is an essential step for genomic DNA sequencing. For an ideal amplification method, the whole genome should be amplified with high fidelity and uniformity. Conventional whole-genome amplification (WGA) methods, including multiple displacement amplification (MDA) and multiple annealing and looping based amplification cycles (MALBAC) use synthetic random oligonucleotides to amplify the genomic DNA^17,18^. However, such random primers have some disadvantages, such as normally featuring certain sequence bias, which accumulates during the amplification process.

Various amplification methods have been developed in which primase is used to synthesize primers, rather than using random primers^14,19^, which is called primase-based whole-genome amplification (pWGA). Using bacteriophage T7 gp4 and T4 replisome, the yield of pWGA was shown to reach over 10^3^-fold amplification. A new method called TruePrime, which uses the *Thermus thermophilus* primase PrimPol to synthesize DNA primers, has also been used in whole-genome DNA amplification; it has been reported to have high amplification activity and low sequence bias^20^. However, such primases recognize a specific sequence for primer synthesis, which might also lead to amplification bias.

In this study, we demonstrate that TtDnaG2 can synthesize long RNA primers with low initiation specificity at 70 °C in the presence of a denatured double-stranded DNA template. It can also be used in whole-genome amplification with high uniformity, and preferentially amplifies circular DNA rather than linear DNA.

## Results

### Bioinformatic analysis of TtDnaG2 revealed the basis of its special priming activity

**Figure 1:**
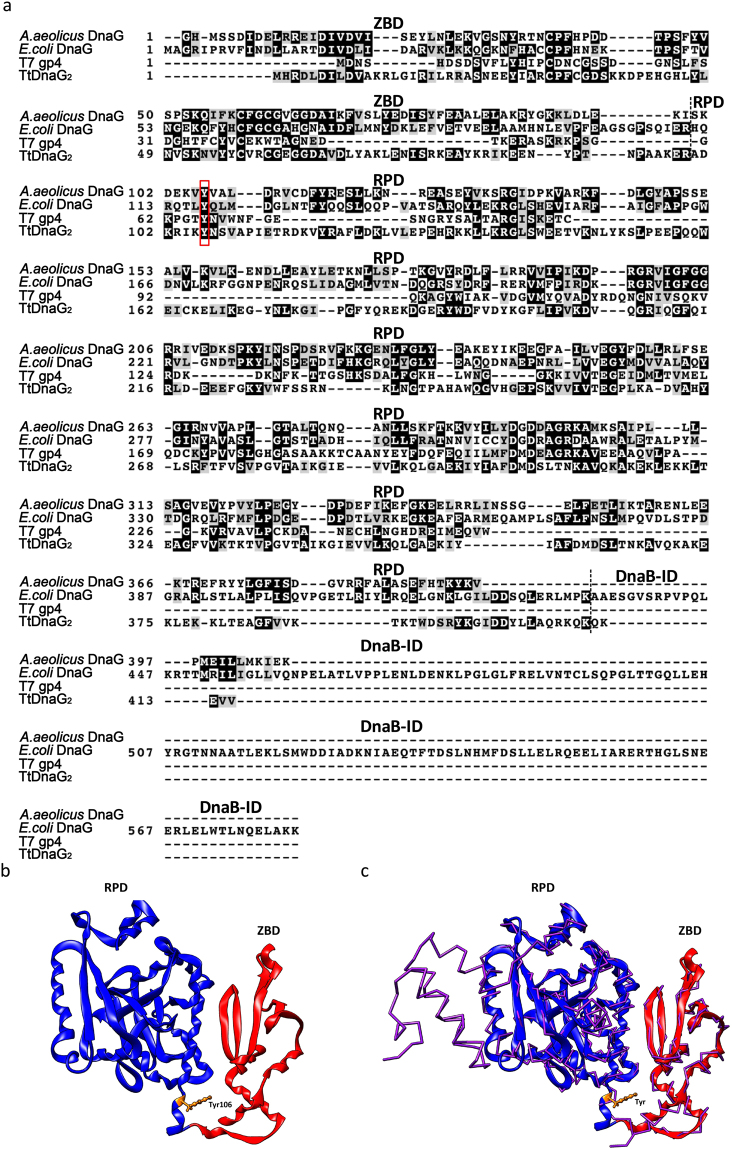
Sequence and structural analysis of TtDnaG2. (**a**) Protein sequence alignment of homologous proteins. The four primases all have the ZBD and RPD domains. (**b**) Structure of TtDnaG2. The position of Tyr106 is coloured yellow. (**c**) Structure of TtDnaG2 and *A*. *aeolicus* DnaG catalytic domain; *A*. *aeolicus* DnaG is displayed as a trace of the backbone. The position of Tyr106 in TtDnaG2 is coloured yellow and the position of Tyr106 in *A*. *aeolicus* DnaG is merged with that of TtDnaG2.

Using structural and functional information on *E*. *coli* DnaG and bacteriophage T7 primase gp4, we deduced the corresponding domains and the conserved catalytic centre of TtDnaG2 (Tyr106). We found that TtDnaG2 has an N-terminal ZBD and RPD^16^, which is consistent with *E*. *coli* primase DnaG. However, it lacks DnaB-ID (Fig. 1a). We then modelled the 3D structure of TtDnaG2 using I-TASSER. By analysing this structure, we found that TtDnaG2 adopts a compact conformation (Fig. 1b). According to our prediction, the structure of TtDnaG2 has a high TM-score with *Aquifex aeolicus* primase DnaG (Fig. 1c), which represents high structural similarity. A previous study proved that *A*. *aeolicus* primases adopt a compact conformation, in which the conserved residues in the ZBD are far away from the active sites of the RPD^21,22^, which decreases the specificity of initiation and restricts the primer length. According to our predicted structure, TtDnaG2 also has a compact conformation, in which the conserved residue Tyr106 in the ZBD is far away from the active site of the RPD. This may explain why the product of TtDnaG2 is long, it may also explain why it has low site specificity with all templates previously tested^16^. However, in *T*. *tengcongensis*, the main primase is TtDnaG, which is more like a conventional primase, while the special features and potential physiological function of TtDnaG2 are unknown.

### TtDnaG2 initiates RNA primer synthesis on a denatured double-stranded template

**Figure 2:**
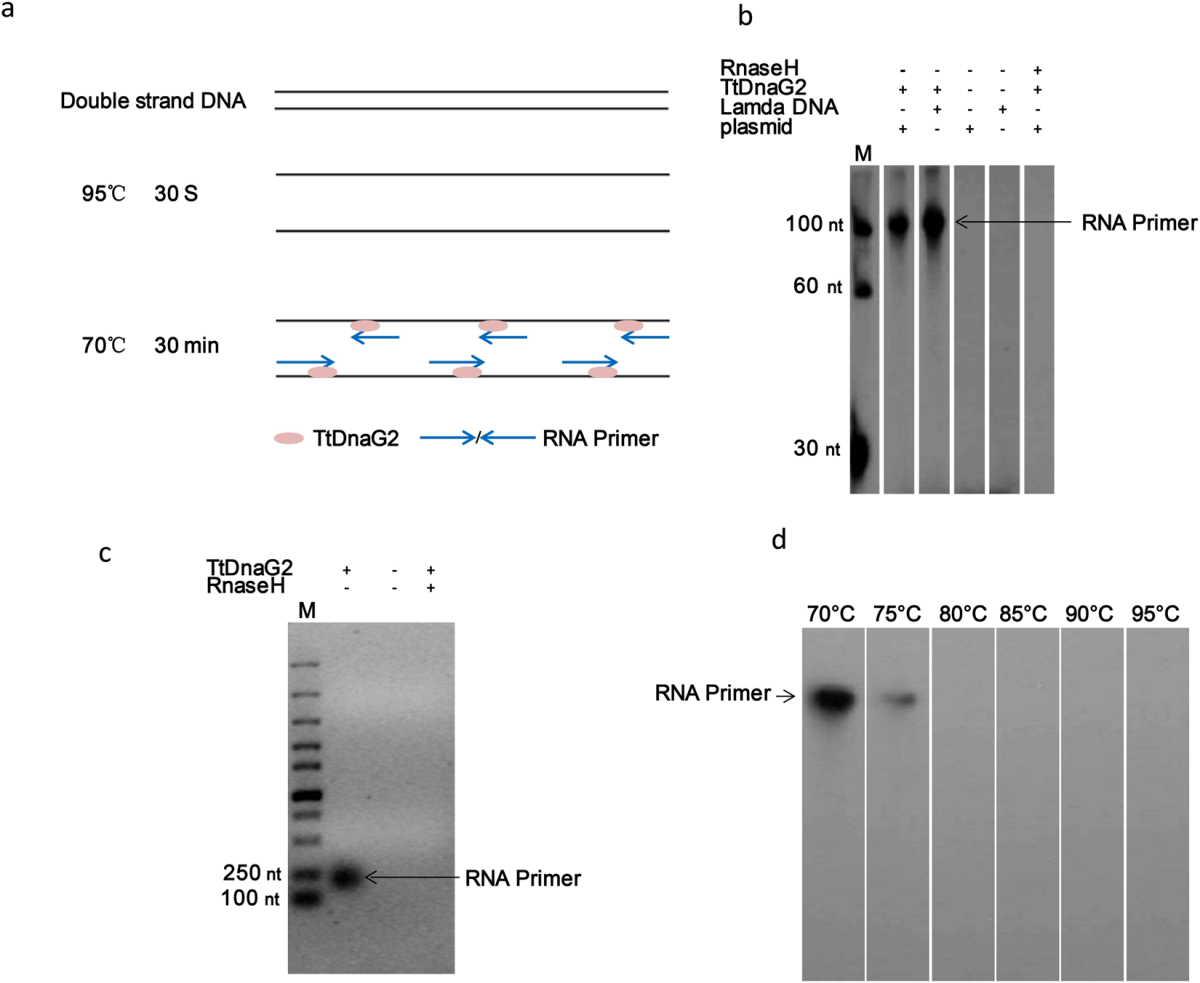
Synthesis of RNA primers with double-stranded DNA template. (**a**) Scheme of the priming process. (**b**) RNA primers synthesized under different conditions. The sample was denatured at 95 °C for 30 seconds and then incubated at 70 °C for RNA primer synthesis ([α-^32^P] ATP was incorporated) with (+) or without (−) TtDnaG2, lambda DNA template, plasmid template. To test whether the labelling product was RNA, the final products were treated with (+) or without (−) RNaseH. The grouping of gels was cropped from the same gel. (**c**) Agarose electrophoresis image of RNA primers; the same as (**b**), except the amplification template was plasmid DNA. (**d**) RNA primers synthesized at different temperatures; the same as (**b**), except for the various temperatures for RNA primer synthesis and that the templates are plasmids. The grouping of gels was cropped from the same gel. The RNA primers synthesized by TtDna2 are indicated by arrowheads. Numbers on the left of each panel indicate the size of α-^32^P-labelled ssDNA markers.

TtDnaG2 does not has apparent template specificity for primer synthesis^16^, in contrast to other known primases, which prefer to initiate primer synthesis at specific DNA sequences^23,24^. We here explore whether it can be used in primase-based whole-genome amplification. A previous study revealed that TtDnaG2 can synthesize RNA primers at a wide range of temperatures on an M13 single-stranded template, as well as other short synthetic DNA oligonucleotides^16^. First, we tested whether TtDnaG2 can synthesize RNA primers on denatured double-stranded DNA. We used the plasmid pET28a and lambda DNA as templates; after denaturing these two double-stranded templates at 95 °C for 30 s, the RNA primers were synthesized at 70 °C for 30 min (Fig. 2a, Supplementary Fig. 8b) in the presence of [α-^32^P]ATP. The RNA products were then analysed by autoradiography. As shown by the gel (Fig. 2b), TtDnaG2 can synthesize RNA by using the denatured double-stranded DNA as a template, and the RNA products are longer than 100 nucleotides. This indicates that TtDnaG2 can withstand 95 °C for 30 s. After heating at 95 °C for 30 s, we also tested the reaction temperatures of 75 °C, 80 °C, 85 °C, 90 °C, and 95 °C for 30 min. In contrast to the findings in a previous study with single-stranded templates, we found that the synthesis of primers on a double-stranded template could hardly occur at temperatures higher than 75 °C (Fig. 2d, Supplementary Fig. 8a). Specifically, in the study of Li *et al*., TtDnaG2 could synthesize primers on a single-stranded template at 85 °C. To explain this difference, we propose that, after being heated at 95 °C for 30 s, TtDnaG2 is denatured at temperatures higher than 75 °C for 30 min. When 50 ng of double-stranded plasmid is used, after being heated at 95 °C for 30 min and primer synthesis occurring at 70 °C for 30 min, according to the results of agarose gel electrophoresis, the primers can be seen (Fig. 2c). In addition, differing from the primers synthesized in the reaction with short synthetic DNA oligonucleotides, the length of the synthetic primers is mainly greater than 100 nucleotides.

### TtDnaG2 and *Bst*3.0 can be used in rolling circular amplification

**Figure 3:**
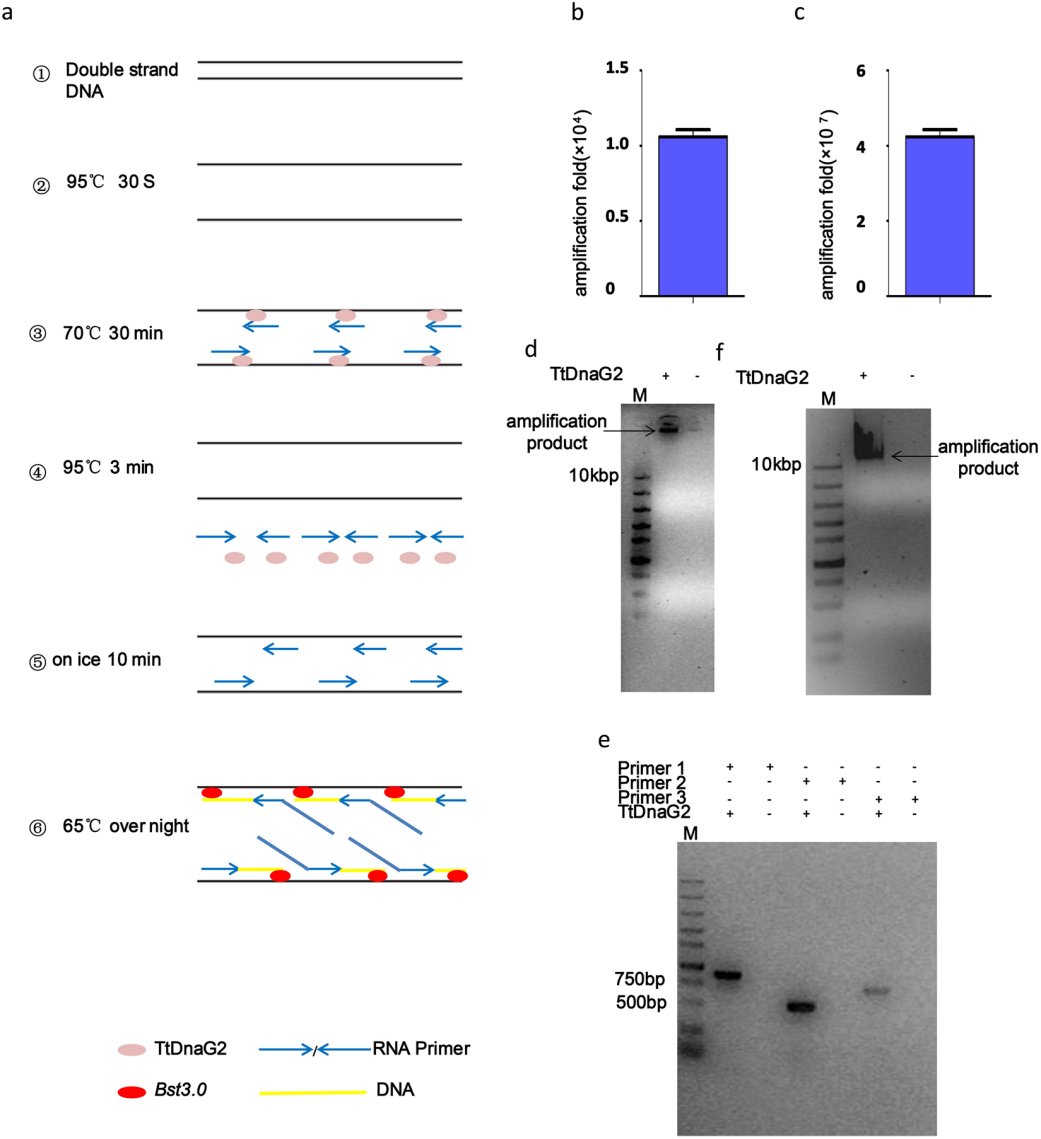
RNA primer synthesis and amplification of DNA. (**a**) Scheme of priming process and amplification. (**b**) Efficiency of circular plasmid amplification. The sample was for the first-round reaction; the template DNA is 2.5 ng. (**c**) Efficiency of circular plasmid amplification. The sample was for the second-round reaction, for which the amplification products of the first-round reaction were used as a template for amplification again. The initial template DNA was 2.5 pg. (**d**) Agarose electrophoresis image of amplification product. The sample was denatured at 95 °C for 30 seconds and then incubated at 70 °C for RNA primer synthesis with (+) or without (−) TtDnaG2. At the amplification step, the mixture was incubated on ice for 10 minutes, followed by adding *Bst*3.0 and incubating at 65 °C overnight. Circular plasmid was used as template. (**e**) Agarose electrophoresis image for checking the PCR results. TtDnaG2 is used in the amplification reaction; primers 1, 2, and 3 are used for checking the PCR results. (**f**) Agarose electrophoresis image of amplification product synthesized by phi29. The sample were denatured at 95 °C for 30 seconds and then incubated at 70 °C for RNA primer synthesis with (+) or without (−) TtDnaG2. At the amplification step, the mixture was mixed with “mixture B” which was mentioned in “Methods”, followed by incubation of the mixture at 95 °C for 3 min, after which it was placed on ice for 10 min. Finally, phi29 was added, followed by incubation at 30 °C overnight. Circular plasmid was used as template.

As TtDnaG2 has special characteristics that make it recognize any DNA sequence that we tested^16^, we further explored whether its RNA product can act as a primer in DNA isothermal amplification. We used the plasmid pET28a as a template and carried out rolling circular amplification. For this, we used the DNA polymerase *Bst*3.0, which has high amplification and displacement activities, to extend the DNA strand beyond the RNA primer. We designed the reaction to proceed in two steps. At the priming step, we added 500 μM dNTP to the reaction system and carried out the reaction at 95 °C for 30 s and 70 °C for 30 min. After the priming step, we changed the temperature to 95 °C for 3 min, to abolish the priming activity of TtDnaG2, and dropped from the template to provide a template for DNA polymerase. Then, we put the reaction system on ice for 10 min, so that the RNA primers could anneal to the templates again. Subsequently, we added the enzyme *Bst*3.0 to the reaction system and kept the temperature at 65 °C overnight for amplification (Fig. 3a). We detected the product of this reaction by agarose gel electrophoresis; the product was found to be very large, which is consistent with our speculation that it is a large DNA molecule with many branches (Fig. 3b). We used at least 2.5 ng of template in the 50-µl reaction system and obtained 26.5 µg of DNA on average, which represented 1.06 × 10^4^-fold amplification (Fig. 3c). When we adopted a two-round amplification strategy, we used 2.5 pg of plasmid template, which led to amplification efficiency of 5.1 × 10^7^ fold on average (Fig. 3d). Then, we tested the amplification products by PCR and sequencing. We designed three pairs of primers to amplify three fragments on pET28a, with the results indicating that the three fragments were all sequences of pET28a (Fig. 3e).

Next, we applied the DNA polymerase phi29, which has strand displacement activity and is widely used in whole-genome amplification^25–27^. As phi29 DNA polymerase and TtDnaG2 cannot work in the same buffer, we needed two reaction systems to amplify DNA. After the priming step, the mixture was added to the phi29 amplification system. In our study, phi29 could also extend the DNA strand following RNA primer synthesis by TtDnaG2 when we used 5 ng of plasmid pET28a as a template (Fig. 3f). As the elongation activity of phi29 is lower than that of *Bst*3.0, the amplification products of phi29 are shorter than those of *Bst*3.0. Our data indicate that TtDnaG2 can be used for efficient primase-based DNA amplification.

### TtDnaG2 and *Bst*3.0 can be used for amplifying circular DNA mixed with linear DNA

**Figure 4:**
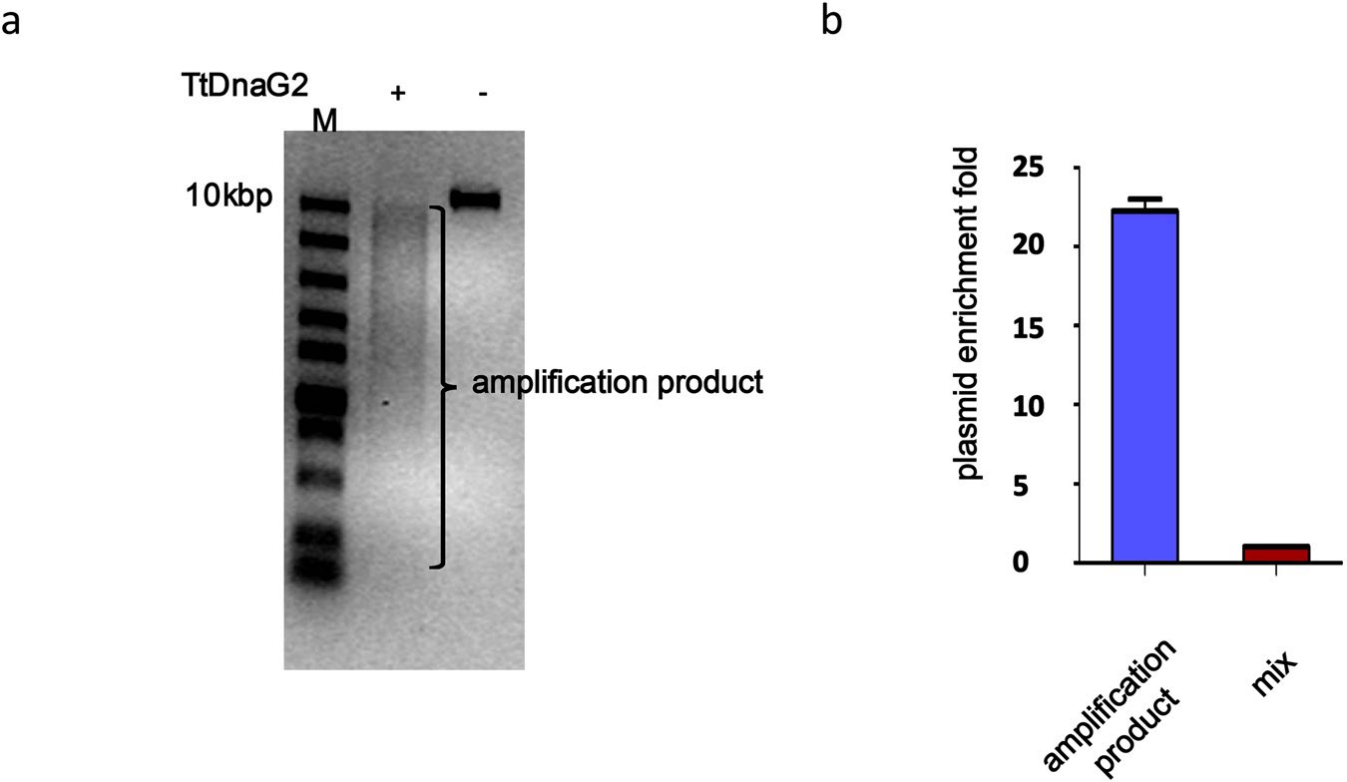
Amplification of circular and linear DNA molecules. (**a**) Amplification product of lambda DNA. Agarose electrophoresis image of amplification product. The sample was denatured at 95 °C for 30 seconds and then incubated at 70 °C for RNA primer synthesis with (+) or without (−) TtDnaG2. At the amplification step, the mixture was incubated on ice for 10 minutes, followed by adding *Bst*3.0 and incubating at 65 °C overnight. Linear lambda DNA was used as template.50 ng of template was used,1/10 of the products were loaded on the gel. (**b**) The relative fold enrichment of plasmid in amplification products of TtDnaG2 and *Bst*3.0; 10 ng of template, involving a mixture of *E*. *coli* genomic DNA and plasmid at a ratio 1000:1, was used. The percentage content of plasmid in the mix was defined as “1” (the red column). The blue column represents the fold enrichment after amplification.

Next, we explore whether the linear template lambda DNA can be amplified with this method (Fig. 4a). As the polymerase *Bst*3.0 can theoretically synthesize a DNA strand without stopping, its amplification product can be longer than lambda DNA. As TtDnaG2 synthesizes primers on a template at random, each primer can be elongated to the terminal of the template, so different primers on the template can produce different products. Our data also indicate that the amplification product of lambda DNA is a mixture of DNA fragments of different lengths, most of which are shorter than lambda DNA (Fig. 4a). As *Bst*3.0 can theoretically elongate without stopping on a circular template, we further explored whether we can amplify linear and circular DNA molecules with different efficiency in a mixture of linear *E*. *coli* genomic DNA and plasmid pET28a. We mixed genomic DNA of *E*. *coli*, which is usually a linear molecule *in vitro*, with the plasmid pET28a. Then, we amplified a mixture with TtDnaG2 and *Bst*3.0, and tested the ratio of plasmid vs. genomic DNA in the amplification products by real-time PCR. We found that the relative amount of plasmid was elevated by 22-fold higher than genomic DNA (Fig. 4b). This indicates that our method might be useful as a tool to efficiently amplify low-copy-number circular DNA when it is mixed with linear DNA.

### TtDnaG2 can be used in whole-genome amplification

**Figure 5:**
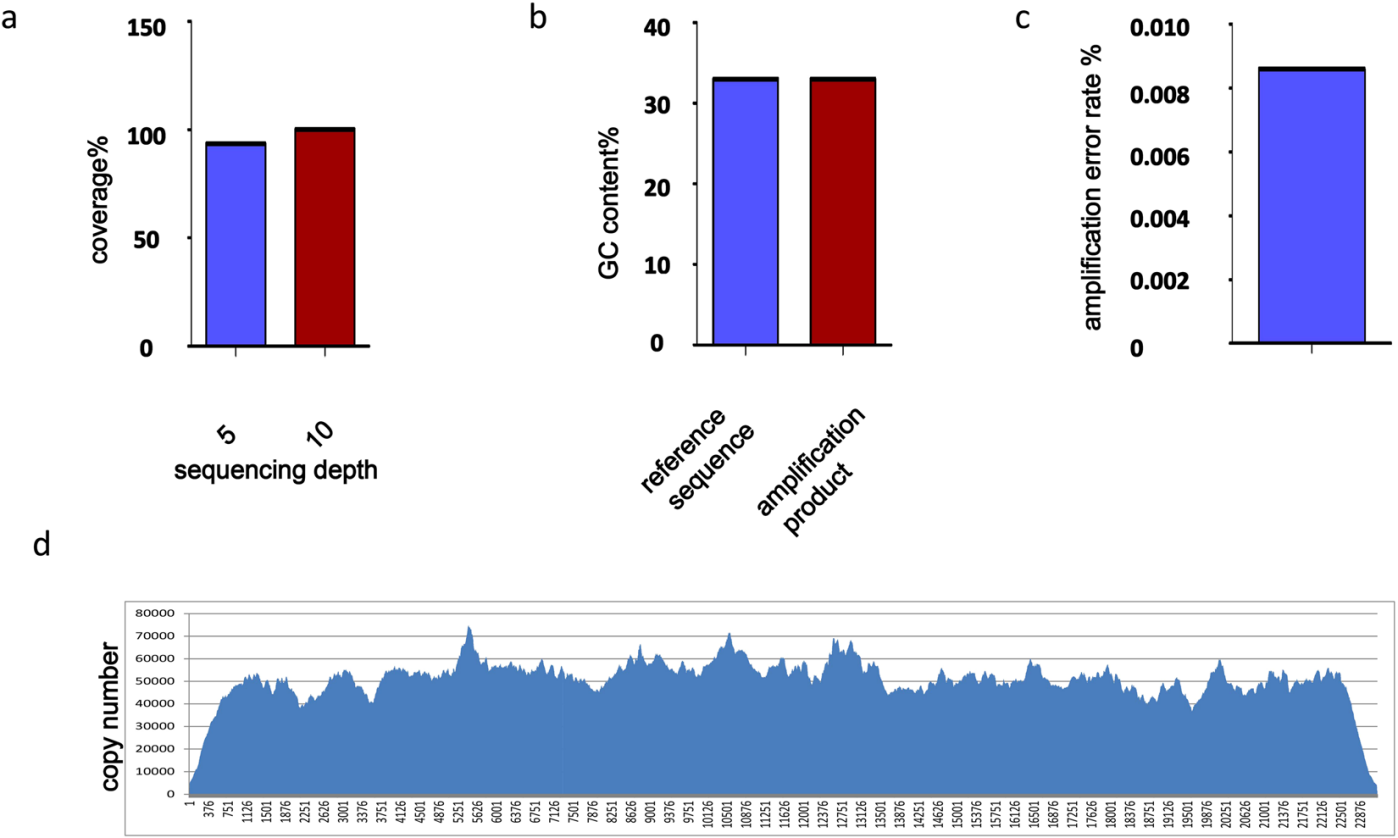
Sequence analysis of amplification products of viral genomic DNA. (**a**) Coverage analysis with reference sequence, at 5- and 10-fold sequencing depth. (**b**) GC contents of amplification products and reference sequence. (**c**) Amplification error rate; two single-nucleotide mutations were found. (**d**) Copy number variation analysis of amplification products.

To test whether our method can be used in whole-genome amplification, we used the enzymes TtDnaG2 and *Bst*3.0 to amplify the genomic DNA of a virus newly discovered in *Sulfolobus solfataricus*. We use 5 ng of genomic DNA as a template for amplification and sequenced the product using next-generation sequencing technology. To determine the amplification quality, we analysed the coverage breadth, GC content, copy number variation (CNV), and amplification error rate. To analyse the coverage of the amplification product, we used the software Burrows–Wheeler Aligner (BWA). We found that, when the sequencing depth was 5- and 10-fold, the coverage was 93.6% and 100%, respectively (Fig. 5a). The GC content was 33%, which is consistent with the reference sequence (Fig. 5b). For the detection of CNV, we used the software Integrative Genomics Viewer (igv_2.3.88), which showed that the CNV is low (Fig. 5d). By single-nucleotide polymorphism analysis, we only found 2 nucleotides that differed from the reference sequence, so the error rate of amplification was 0.0086% (Fig. 5c). Accordingly, we can thus amplify the genomic DNA of this virus effectively with the combination of TtDnaG2 and *Bst*3.0.

## Discussion

Primases usually synthesize RNA primers on a single-stranded DNA template *in vivo*. In the priming step of DNA replication, double-stranded template is unwound by helicase^28,29^, and ssDNA binding protein is needed to stabilize ssDNA. Then, primases can synthesize RNA primers. In our study, we found that the primase TtDnaG2 can synthesize RNA primers at high temperature *in vitro*, when double-stranded DNA is denatured. The priming process does not require helicase and ssDNA binding protein. Consistent with the previous findings^16^, the priming product of TtDnaG2 is long, unlike most reported primases. The RNA primers were longer than 100 nucleotides in our study and the priming activity was high. Another characteristic of TtDnaG2 is that it has low site bias. A previous sequence analysis indicated that TtDnaG2 lacks motif IV in RPD, which is consistent with the structure of T7 bacteriophage gp4^16,30^. gp4 synthesizes RNA short primers at specified sites, so the lack of motif IV in RPD of TtDnaG2 may not be the reason why it has low site bias and can synthesize long RNA products^31^. The modelled 3D structure of TtDnaG2 indicates that it adopts a compact conformation, in which the active sites that control the specificity and length of primers are sequestered away from the synthesis domain. This may explain why TtDnaG2 can synthesize long RNA primers with low sequence bias.

Primase is thought to be promising in whole-genome amplification to overcome some shortcomings in whole-genome amplification^19^. In this context, the fact that TtDnaG2 can synthesize RNA primers at high temperature *in vitro*, without the need for helicase and ssDNA binding protein, suggests its potential in whole-genome amplification. Upon combining TtDnaG2 with *Bst*3.0 or phi29, which have high strand displacement activity and can use RNA primers to initiate amplification, we amplified the plasmid at a rate of 5.1 × 10^7^-fold. The reaction process can be summarized as follows: In the primer synthesis step, double-stranded DNA is denatured by heating at 95 °C. Then, TtDnaG2 synthesizes RNA primers at 70 °C, after which TtDnaG2 synthesis is abolished by exposure to 95 °C for 3 min. In the elongation step, *Bst*3.0 or phi29 elongates a new DNA strand with RNA primers. Our data also indicate that *Bst*3.0 and phi29 can use RNA primers to elongate DNA strands and may displace downstream RNA encountered. Compared with previously reported T4 and T7 pWGA, which need ssDNA binding protein or other proteins, our method is simpler as helicase or ssDNA binding protein is not required. Compared with TruePrime, TtDnaG2 has been proved to have low sequence bias, which may lead to lower amplification bias. The amplification of genomic DNA and analysis of sequencing results indicate that it has high amplification activity, 100% coverage, the CNV is low, and the error rate is lower than 1/10^4^.

To reduce the nucleotide error rates in the process of amplification, we used high-fidelity DNA polymerases phi29 and Pfu after priming to amplify DNA. However, we did not observe DNA amplification with the combination of Pfu DNA polymerase and TtDnaG2. Phi29 can use RNA primers generated by TtDnaG2 to amplify DNA, but it cannot work in the same buffer with TtDnaG2. We need a buffer exchange between priming and DNA amplification. *Bst*3.0 is a high-processive polymerase but has no 3′ → 5′ exonuclease activity. In this study, we found only two nucleotides that differed from the reference sequence by *Bst*3.0 amplification. Given that the virus genome is 23,219 bp, the error rate is 2/23,219. Accordingly, the error rate of *Bst*3.0 is lower than 1/10^4^ in our reaction system, and it is comparable to that for Phi29 (error rate of 1/10^4^–1/10^6^)^32^. Moreover, TtDnaG2 and *Bst*3.0 work well in the same reaction buffer. Thus, it would be advantageous to combine TtDnaG2 with *Bst*3.0 to amplify genomic DNA. We believe that this method could contribute greatly to obtaining complex genomic information accurately.

In the amplification of a mixture of linear *E*. *coli* genomic DNA and circular plasmid, the amplification efficiency of plasmid was 22-fold higher than that of the linear DNA. *Bst*3.0 can theoretically elongate a strand continuously on a circular DNA template, while the linear DNA in an experiment normally reaches 300 Kbp in length at most. This results in high amplification efficiency for circular templates and lower efficiency for short linear ones. Theoretically, when the linear DNA template is sufficiently long, the amplification difference is small; however, the shorter the linear DNA template, the larger the difference. Long primers synthesized by TtDnaG2 also overcome the limitation of *Bst*3.0 that it cannot use short random primers at 65 °C. These features make the method a potential tool to amplify low-copy-number circular DNA such as plasmid DNA or circular viral DNA from a whole DNA sample where the circular DNA might not be efficiently amplified and the information may be lost in the subsequent sequencing analysis.

## Methods

### Expression and purification of TtDnaG2

Upon the transformation of *E*. *coli* competent cells Rosetta (DE3) with the plasmid pET28a-TtDnaG2, the cells were cultured in Luria-Bertani (LB) medium with 50 µg/mL kanamycin at 37 °C until they grew to OD_600_ 0.4 to 0.6, after which isopropyl-β-D-thiogalactopyranoside (IPTG) was added to a final concentration of 0.5 mM. The cells were then induced to express the protein at 37 °C for 3 h. After culture, the cells were harvested by centrifugation at 5000 rpm for 30 min, followed by resuspension of the pellet in lysis buffer [0.3 M NaCl, 10 mM imidazole and 50 mM sodium phosphate buffer (NaH_2_PO_4_/Na_2_HPO_4_, pH8.0)] and then ultrasonication for 50 min. To obtain soluble protein, the lysates were centrifuged at 10,000 rpm for 30 min, the pellet was discarded, and the supernatant was incubated at 70 °C for 30 min, followed by another session of centrifugation under the same conditions. The pellet was then discarded and the supernatant was passed through a 0.45-µm filter. Next, the protein TtDnaG2 was purified by affinity chromatography with a Ni_2_-nitrilotriacetic acid–agarose column and gel filtration chromatography with a Superdex 200 10/300 GL column. The purified protein was detected by SDS-PAGE and then concentrated with Amicon Ultrafra-15 concentrators, after which it was stored in 10% glycerol at −70 °C.

### RNA primer synthesis assay

The 25-µL priming system contained 50 mM HEPES (pH7.5), 10 mM dithiothreitol, 100 mM potassium glutamate, 5 mM magnesium acetate, 100 μM NTP mixture, 1 μCi [α-^32^P]ATP, 50 ng of plasmid pET28a or lambda DNA template, and 1 µM TtDnaG2. The reaction mixture was incubated at 95 °C for 30 s, followed by 70 °C for 30 min, after which 50 μL of 3 M sodium acetate (pH5.2) was added to the system to stop the reaction. Then, the RNA primer was precipitated with 225 μL of cold ethanol and 40 µg of glycogen at −20 °C overnight, followed by centrifugation at 13,300 rpm for 30 min. Next, the pellet was washed twice with 75% ethanol, dried at room temperature for 15 min, and dissolved in 20 μL of loading buffer containing 98% formamide, 0.025% bromophenol blue, 0.025% xylene cyanol FF, and 10 mM Ethylenediaminetetraacetic acid (EDTA) (pH8.0). After heating the sample at 95 °C for 3 min, a 10-µL sample was loaded onto denaturing polyacrylamide sequencing gel containing 15% acrylamide–bisacrylamide (39:1), 8 M urea, 0.05% ammonium persulfate and 0.05% TEMED. The gel was then run at 300 V for 2 h. After electrophoresis, the gel was analysed by autoradiography with X-ray film.

### DNA amplification assay

For amplification with TtDnaG2 and *Bst*3.0, in the primer synthesis step, the 50-µL reaction system contained 50 mM HEPES (pH7.5), 10 mM dithiothreitol, 100 mM potassium glutamate, 5 mM magnesium acetate, 100 µM NTP mixture, 500 µM dNTP mixture, and 2.5 ng of plasmid pET28a template (or 50 ng of lambda DNA or a 10-ng mixture of genomic DNA and plasmid). The synthesis programme started with denaturation at 95 °C for 30 s, followed by synthesis at 70 °C for 30 min. At the amplification step, the mixture was incubated at 95 °C for 3 min, then on ice for 10 min, followed by adding 2 µL of *Bst*3.0 and incubating at 65 °C overnight. For two-round amplification of 2.5 pg of plasmid DNA, we used a 20-µL reaction system in the first round, with an amplification time of 3 h. In the second round, we added a 30-μL reaction mix [50 mM HEPES (pH7.5)], 10 mM dithiothreitol, 100 mM potassium glutamate, 5 mM magnesium acetate, 100 μM NTP mixture, 500 μM dNTP mixture, and 1 µM TtDnaG2), after which the priming and amplifying steps were carried out.

For amplification with TtDnaG2 and phi29, in the primer synthesis step, the 1-µL reaction mixture (mixture A) contained 50 mM HEPES (pH7.5), 10 mM dithiothreitol, 100 mM potassium glutamate, 5 mM magnesium acetate, 100 μM NTP mixture, and 5 ng of plasmid pET28a. The synthesis programme started with denaturation at 95 °C for 30 s, followed by synthesis at 70 °C for 30 min. At the amplification step, mixture A was first mixed with mixture B (9 µL), which contained 1 µL of 10* reaction buffer for phi29 DNA polymerase, 250 µM dNTP mixture, and 5 ng of plasmid pET28a, followed by incubation of the mixture at 95 °C for 3 min, after which it was placed on ice for 10 min. Finally, 1 µL of phi29 was added, followed by incubation at 30 °C overnight.

### qPCR assay of amplification products and calculation of fold enrichment

The primers designed for genomic DNA qPCR were as follows: E-f (CCAGTGGTCGCATCATCGTTA), E-r (CCATTATCTCGGTGGTAGGTG); those for plasmid pET28a were: P-f (CGACATATCGGATTGTCCCTA), P-r (TCGGCCAGATCGTTATTCAGT). The fold enrichment was calculated using the following formula: ΔCt = Ct(P) − Ct(E), −ΔΔCt = − [ΔCt(amplification products) − ΔCt(control)]. The fold enrichment was 2^−ΔΔCt^.

### PCR assay

We designed three pairs of primers to amplify the amplification products of pET28a:

F1: GGTCAGACTAAACTGG, R1: GGAGAAAACTCACCGA;

F2: GTAGTGGGATACGACG, R2: GCGTTTCCAGACTTTAC;

F3: GGTTGCATTCGATTCC, R3: CCCTGATAGACGGTTT.

The amplification reaction mixture was diluted 1:100, after which 1 μL was added to the PCR reaction mix. We used Takara PrimeSTAR MAX to clone the three fragments.

### Agarose gel electrophoresis assay

Here, 1.2% agarose gel was prepared with TAE buffer containing 40 mM Tris-acetate and 2 mM EDTA. Next, our sample and the DNA ladder were loaded on an agarose gel and electrophoresed at 100 V for 25 min. After staining with ethidium bromide, the result was analysed by UV imaging.

### Quantification of the DNA output

The sample was precipitated with three volumes of precooled ethanol and stored at −20 °C for 2 h. It was then centrifuged at 13,300 rpm and 4 °C, after which the supernatant was discarded and the remainder was washed twice with 70% ethanol. Following drying at room temperature, the sample was dissolved with sterile water. The DNA was then quantified with a spectrometer and the output was calculated.

### Sequencing of amplification product

After amplification of the whole-genome DNA, the DNA sample was sequenced by Novogene. The three short PCR products were sequenced by TSINGKE, with sequencing primers F1, F2, and F3.

### Sequence coverage, single nucleotide polymorphism (SNP), and CNV analysis

The sequences of the PCR products were subjected to a Blast search by the software A Plasmid Editor (ApE). The coverage of whole-genome amplification products was analysed by the software BWA, while CNV was analysed by the software Integrative Genomics Viewer. The SNP was analysed by the software Genome Analysis Toolkit (GATK). The reference sequence of the viral genome was from GenBank (MF144115).

### Data availability

All data generated or analysed during this study are included in this published article and its Supplementary Information files.

## Electronic supplementary material

Supplementary Information
