# Supplementary material for: The application of thermophilic DNA primase TtDnaG2 to DNA amplification
Source: Sci Rep. 2017 Oct 9;7:12809. doi: 10.1038/s41598-017-12241-6 (PMC5634424; doi:10.1038/s41598-017-12241-6)
Supplement: Supplementary file 1 — Supplementary Information [file 41598_2017_12241_MOESM1_ESM.doc]

# Supplementary Information:The application of thermophilic DNA primase TtDnaG2 to DNA amplification

De Zhao^1,2^, Xiuqiang Chen^1,2^, Kuan Li^3^, Yu Fu^1,2*^

^1^State Key Laboratory of Microbial Resources, Institute of Microbiology, Chinese Academy of Sciences (CAS), Beijing, 100101, China

^2^Savaid Medical School, University of Chinese Academy of Sciences, Beijing, 100101, China

^3^State Key Laboratory of Mycology, Institute of Microbiology, Chinese Academy of Sciences (CAS), Beijing, 100101, China

^*^Correspondence: Yu Fu, Institute of Microbiology, Chinese Academy of Sciences, Beijing, 100101, P.R. China

Email: [fuyu@im.ac.cn](mailto:fuyu@im.ac.cn), Tel.: +86-10-64806126, Fax: +86-10-64806126

**
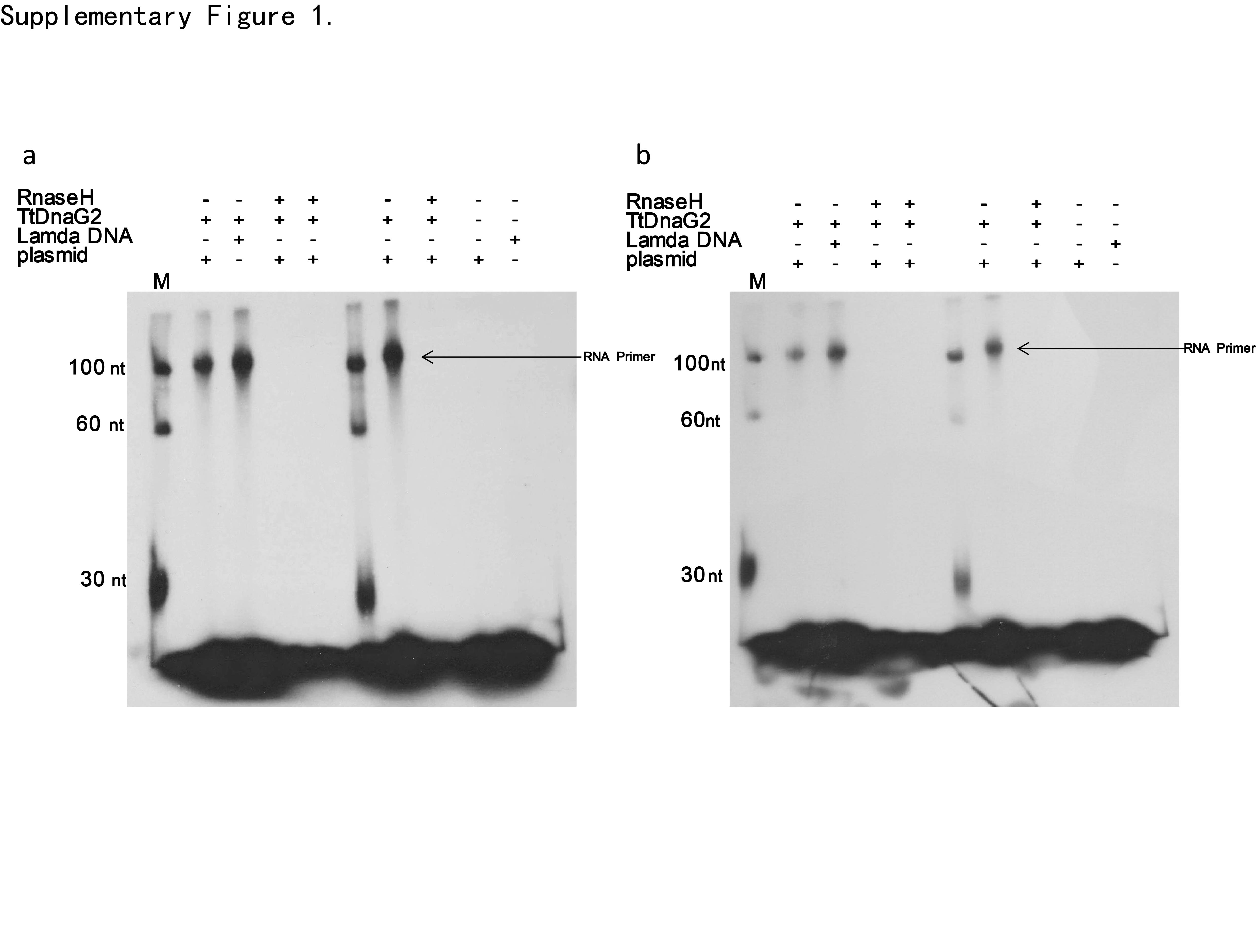
Supplementary Fig. 1. Synthesis of RNA primers. (a)** The full-length gel of Fig.2b. **(b)** The same gel with Supplementary Fig.1a. The exposure time was shorter than that of Supplementary Fig.1a.


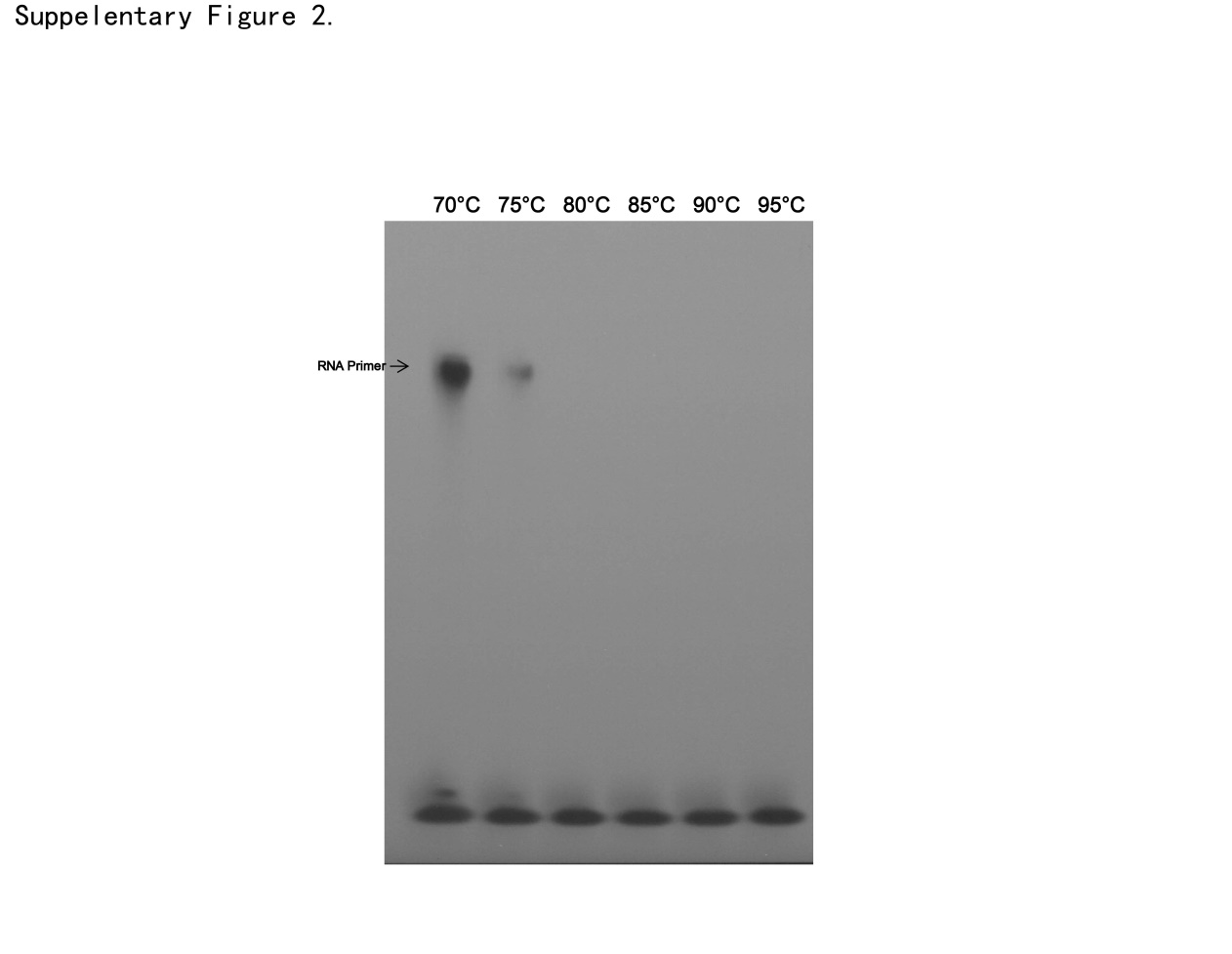


**Supplementary Fig. 2. The full-length gel of Fig.2d.**

**
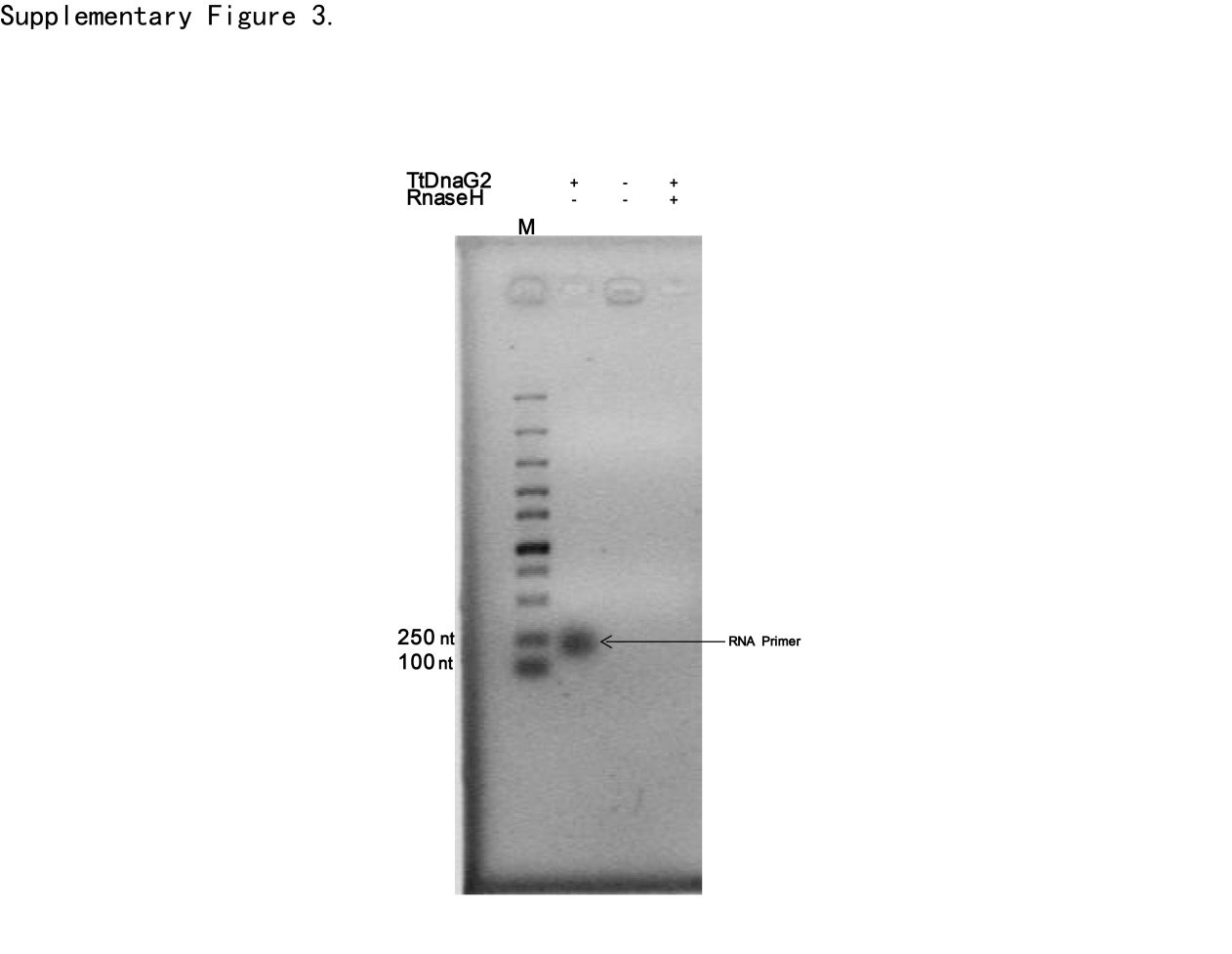
**

**Supplementary Fig. 3. The full-length gel of Fig.2c.**


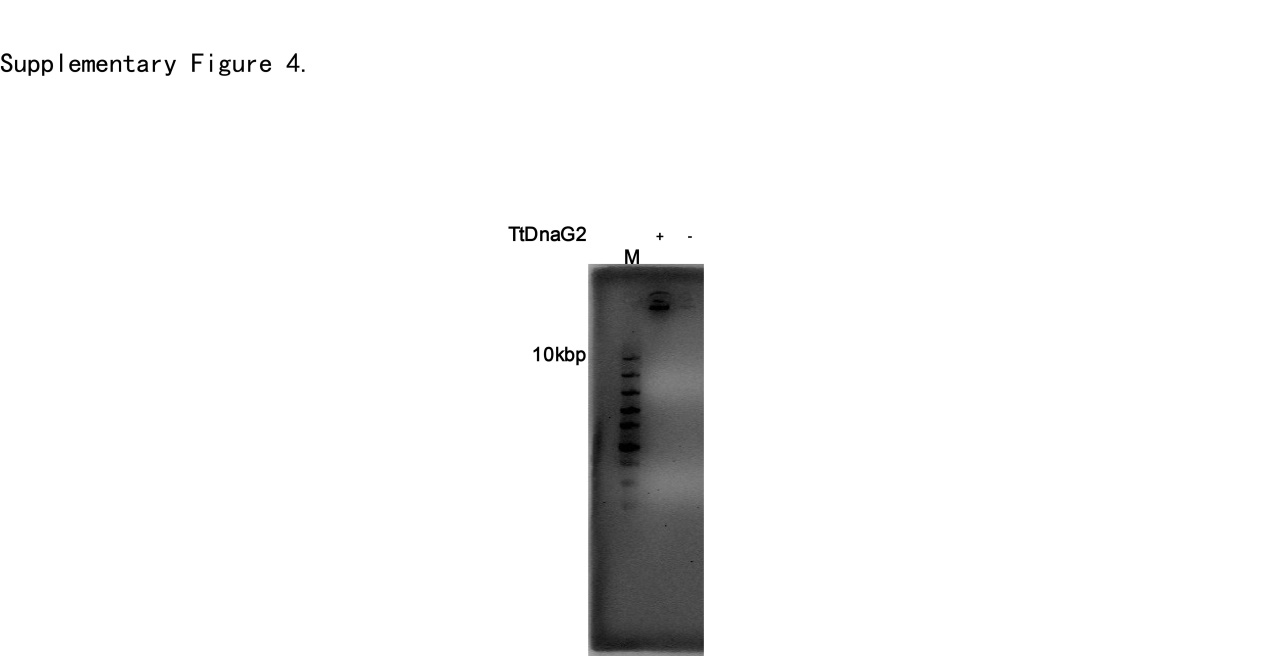


**Supplementary Fig. 4. The full-length gel of Fig.3d.**


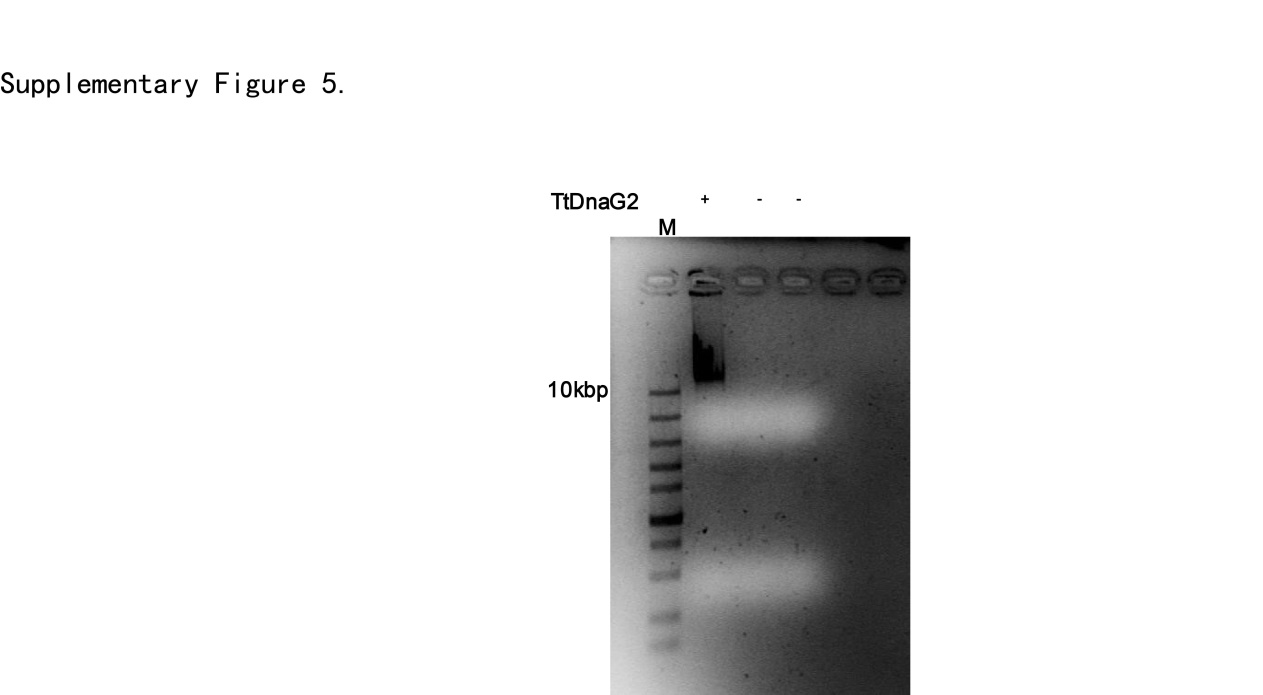


**Supplementary Fig. 5. The full-length gel of Fig.3f.**
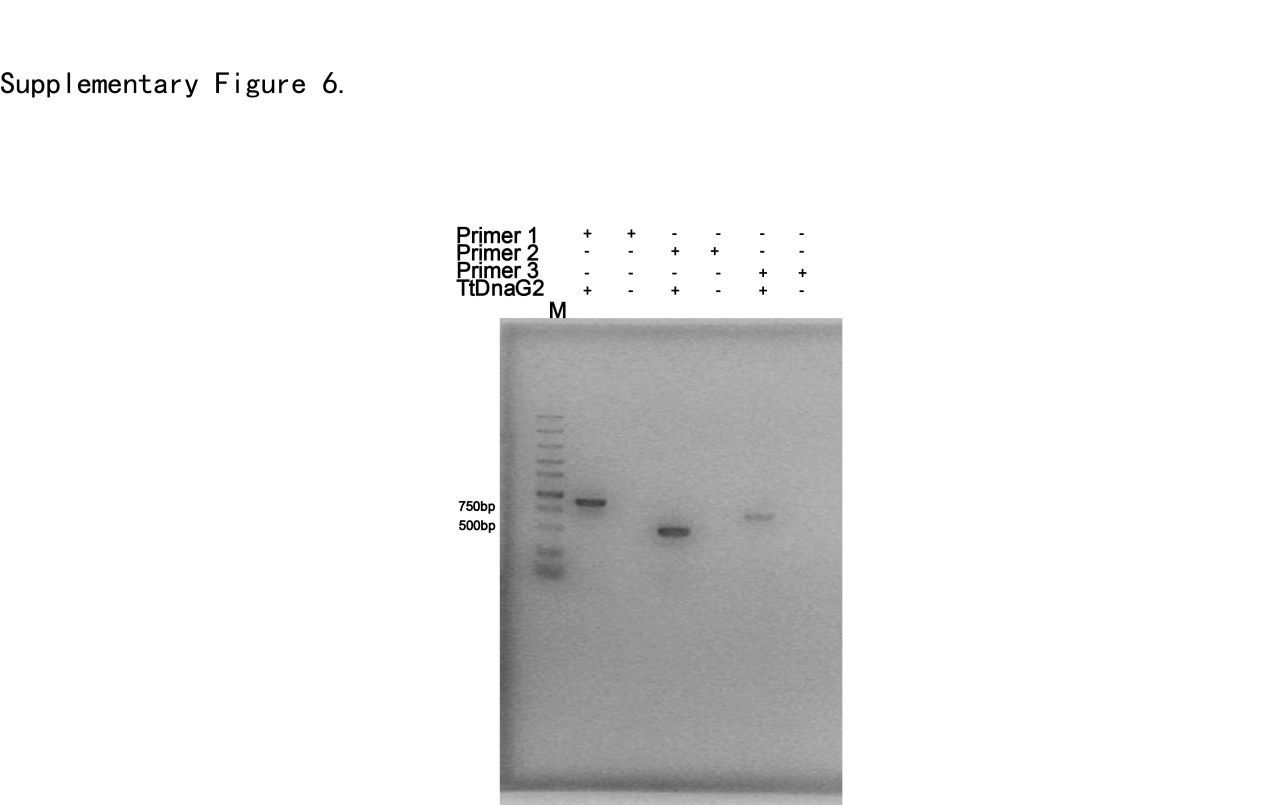


**Supplementary Fig. 6. The full-length gel of Fig.3e.**


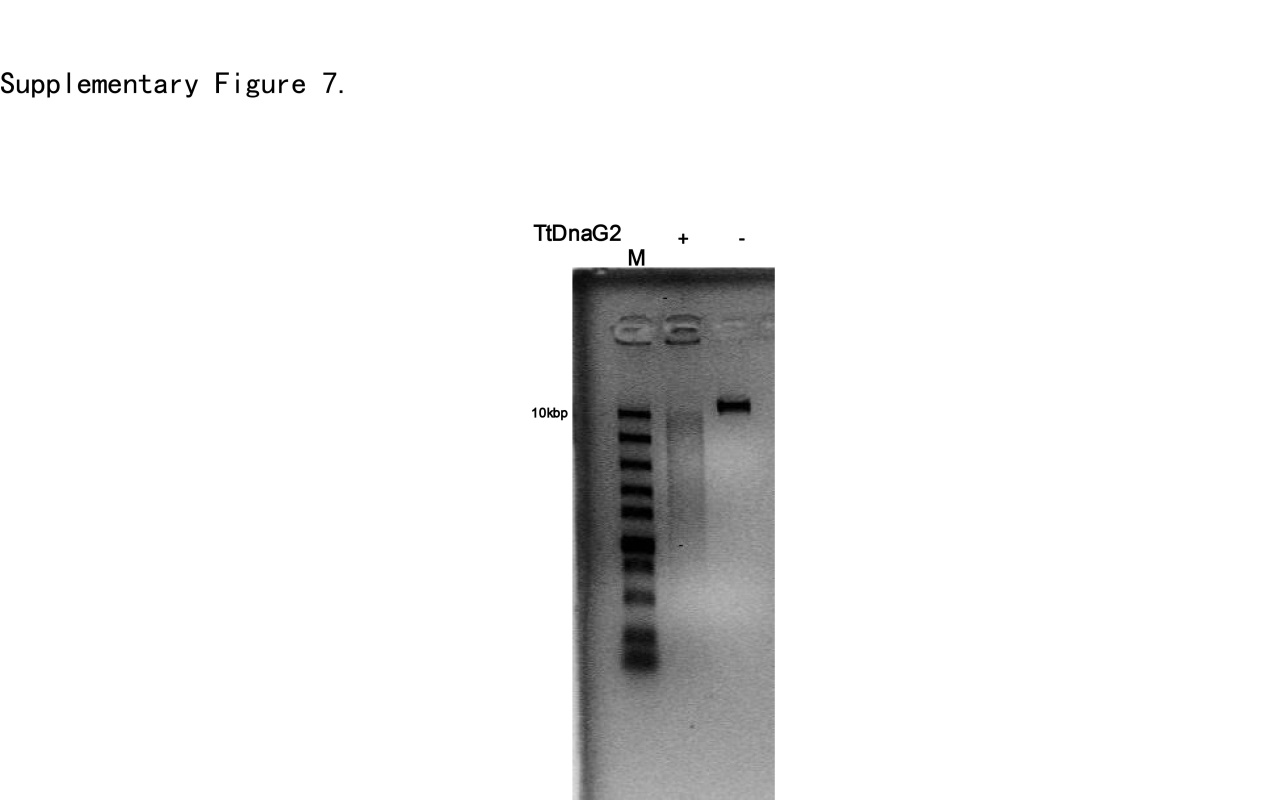


**Supplementary Fig. 7. The full-length gel of Fig.4a.**


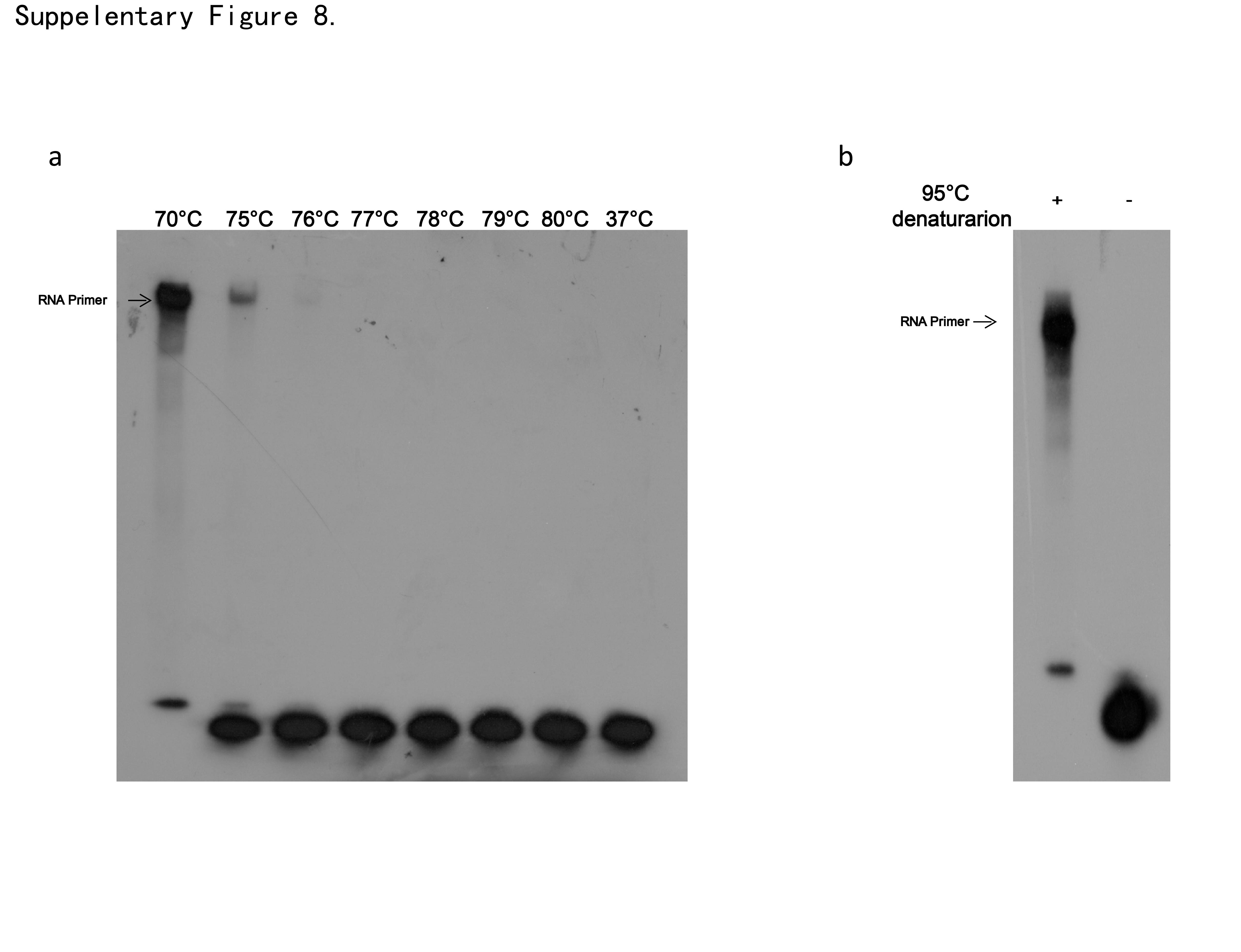


**Supplementary Fig. 8. Synthesis of RNA primers with double-stranded under different conditions. (a)** RNA primers synthesized under different conditions.Samples were denaturated at 95°C for 30 seconds,then incubated at different tempretures. The template was plasmid DNA. **(b)** RNA primers synthesized under different conditions.The sample in the left lane was denaturated at 95°C for 30 seconds, that in the right lane was incubated at room tempreture for 30 seconds,then they were all incubated incubated at 70°C for 30 minutes.RNA primers were labelled with ^32^P.
